# The Relationship between Inflammatory Marker Levels and Hepatitis C Virus Severity

**DOI:** 10.1155/2016/2978479

**Published:** 2016-12-20

**Authors:** Qitian He, Quan He, Xue Qin, Shan Li, Taijie Li, Li Xie, Yan Deng, Yu He, Yongbin Chen, Zhifu Wei

**Affiliations:** ^1^Department of Clinical Laboratory, First Affiliated Hospital of Guangxi Medical University, Nanning, Guangxi Zhuang Autonomous Region 530021, China; ^2^Department of Traditional Chinese Medicine, First Affiliated Hospital of Guangxi Medical University, Nanning, Guangxi Zhuang Autonomous Region 530021, China; ^3^Department of Infection Control, First Affiliated Hospital of Guangxi Medical University, Nanning, Guangxi Zhuang Autonomous Region 530021, China

## Abstract

*Background. *Red cell distribution width (RDW) and platelet-lymphocyte ratio (PLR) have been studied in a variety of etiological diseases. We aim to investigate the relationship between RDW and PLR and the severity of hepatitis C virus- (HCV-) related liver disease.* Methods. *We included fifty-two chronic HCV and 42 HCV-related cirrhosis patients and 84 healthy controls. Hematological and virological parameters and liver function biomarkers of HCV-related patients at admission were recorded.* Results. *RDW, RDW-to-platelet (RPR), and 1/PLR values in HCV-related cirrhosis patients were significantly higher than in chronic HCV patients and healthy controls (all *P* < 0.001). The aspartate aminotransferase (AST)/alanine aminotransferase (ALT) ratio (AAR), AST-to-platelet ratio index (APRI), and fibrosis index based on the four factors (FIB-4) scores in HCV-related cirrhosis patients were significantly higher than in chronic HCV patients (all *P* < 0.001). The areas under the curve of the RDW, RPR, and 1/PLR for predicting cirrhosis were 0.791, 0.960, and 0.713, respectively. Bivariate logistic regression analysis showed that RDW could independently predict the presence of cirrhosis in chronic HCV patients.* Conclusions. *RDW, RPR, and PLR may be potential markers for estimating HCV severity.

## 1. Introduction

Hepatitis C virus (HCV) is predominantly transmitted through blood transfusion and vertical and iatrogenic transmission. Chronic HCV is one of the main etiologies for the morbidity of viral hepatitis worldwide that can lead to long-term complications, including HCV-related cirrhosis and hepatocellular carcinoma (HCC) in a proportion of cases [1, 2]. Globally, an estimated 130–170 million people have HCV infection. Its prevalence highly varies with regard to the regional distribution. The majority of HCV infection cases occur in developing countries, while China accounts for the largest number of HCV infection cases in terms of absolute numbers [3].

In liver biopsy, invasive and some contraindications that render it ineffective in the dynamic monitoring of the disease status need to be considered [4, 5]. Several noninvasive methods such as aspartate aminotransferase (AST)/alanine aminotransferase (ALT) ratio (AAR), aspartate aminotransferase-to-platelet ratio index (APRI) [6], and fibrosis index based on the four factors (FIB-4) [7] have been used to assess disease severity. Recently, some parameters obtained from or calculated by the routine blood tests, including the red cell distribution width (RDW), RDW-to-platelet ratio (RPR), and PLR, can serve as independent risk factors in various diseases [8–13]. There are few articles on the relationship between the RDW, RPR, and PLR and hepatitis C in literature. We investigate whether these parameters are useful variables for determining HCV severity.

## 2. Materials and Methods

### 2.1. Patients

Our study included 52 chronic HCV patients and 42 HCV-related cirrhosis patients consistent with internalize standard and who were hospitalized in the First Affiliated Hospital of Guangxi Medical University from June 2012 to April 2016. Chronic HCV was confirmed in patients if they were anti-HCV positive for 6 months or more and also HCV RNA-positive. HCV-related cirrhosis was determined by clinical manifestations, abdominal imaging, surgical findings, and laboratory results. Patients coinfection with hepatitis B/D/G virus, nonalcoholic fatty liver disease (NAFLD), primary biliary cirrhosis (PBC), autoimmune disorders, malignancy (including HCC), acquired immune deficiency syndrome, hematological diseases, atherosclerotic disease, hypertension, cardiac diseases, diabetes mellitus, renal diseases, previous blood transfusions, chronic inflammatory diseases, and infectious diseases were excluded. In our study of patients with chronic HCV, 10 patients had started antiviral treatment before admission; 42 patients did not receive any antiviral treatment. In patients with HCV-related cirrhosis, 4 patients had started antiviral treatment before admission; 38 patients did not receive any antiviral treatment. The data of 84 healthy individuals were also collected as the control group.

### 2.2. Laboratory Analysis

The fasting blood samples for detecting CBCs and biochemical parameters were collected through the elbow vein. The blood samples of CBCs for detecting white blood cell (WBC) count, platelet count, and RDW values were sampled using tubes containing dipotassium EDTA, and the analysis was carried out using a Beckman Coulter LH750/LH780 hematology analyzer (Beckman Coulter Inc., Fullerton, CA, USA) within 1 hour of blood collection. Biochemical parameters were analyzed using an automatic analyzer (Hitachi 7600; Tokyo, Japan). Coagulation indicators were determined using an ACL-TOP700 automatic coagulation analyzer. Demographic data and laboratory results in HCV-related liver cirrhosis patients on admission were extracted. The AAR was obtained by dividing the AST by ALT. The RPR was obtained by dividing the RDW by platelet count. The PLR was obtained by dividing platelet count by lymphocyte count. The PLR was translated into 1/PLR when appropriate. APRI and FIB-4 are calculated using the following formulas: APRI = (AST/Upper  limit  of  normal) × 100/Platelet  (10^9^/L) [6]; FIB-4 = (Age × AST)/[Platelet × (ALT)^1/2^] [7].

This study was approved by the Ethics Committee of the First Affiliated Hospital of Guangxi Medical University.

### 2.3. Statistical Analysis

Mean ± standard deviation of continuous variables were obtained in this study.

Differences in normally and nonnormally distributed data between groups were analyzed using Student's *t*-tests and Mann–Whitney *U* tests, respectively. The chi-square test was used to analyze categorical data. The relationship between the variables was determined using Spearman's correlation analysis. Binary logistic regression analysis was used to evaluate the underlying factors, including age, AAR, APRI, FIB-4, and RDW associated with HCV-related cirrhosis. The capability of RPR in identifying the presence of liver cirrhosis in chronic HCV patients was determined by the receiver operating characteristic (ROC) curve analysis. SPSS 17.0 (SPSS Inc., Chicago, IL, USA) statistical software was used to analyze the data. *P* < 0.05 was considered statistically significant.

## 3. Results

### 3.1. Comparison of Demographic and Laboratory Results between Patients and Controls

Demographic and laboratory results of HCV-infected patients (*n* = 94) and controls (*n* = 84) were shown (Table 1). Gender and age of HCV-infected patients and healthy subjects were matched (all *P* > 0.05). In comparison to the controls, WBC counts, hemoglobin values, and platelet counts were significantly low, whereas RDW, RPR, and 1/PLR values were significantly high in HCV-infected patients (all *P* < 0.001).

### 3.2. Comparison of Demographic and Laboratory Results between Chronic HCV Patients and HCV-Related Cirrhosis Patients

Demography, clinical characteristics, and laboratory parameters of HCV infection patients are presented in Table 2. Fifty-two chronic HCV (36 males and 16 females) cases and 42 patients with HCV-related cirrhosis (29 males and 13 females) anti-HCV-positive and HCV RNA-positive were included. In patients with chronic HCV, 5 patients had splenomegaly, and 47 patients had normal spleen. In patients with HCV-related cirrhosis, 35 patients had splenomegaly, and 7 patients had normal spleen. Splenomegaly in patients with HCV-related cirrhosis was significantly more than that in patients with chronic HCV (*P* < 0.001). The RDW, RPR, and 1/PLR in HCV- related cirrhosis were significantly higher than in chronic HCV patients (all *P* < 0.05). Correlation analysis showed that the RPR had positive correction with APRI and FIB-4 (all *P* < 0.001). The relationship between the PLR and FIB-4 presented a negative correlation (*P* = 0.007).

### 3.3. Relative Risk Factors for Liver Cirrhosis

**Figure 1:**
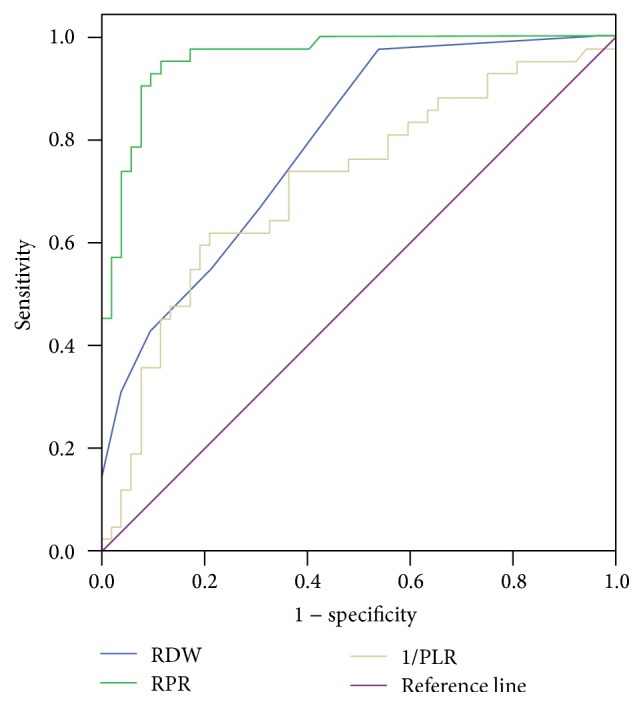
Receiver operating characteristic curve analysis of RDW, RPR, and 1/PLR in identifying the presence of liver cirrhosis in chronic HCV patients.

The diagnostic capacities of RDW, RPR, and 1/PLR in identifying cirrhosis in chronic HCV patients were compared using the ROC curves, and the AUCs of these parameters were found to be 0.791 ± 0.045, 0.960 ± 0.018, and 0.713 ± 0.055, respectively (Figure 1). Potential risk factors, including age, AAR, APRI, FBI-4, and RDW, were investigated using binary logistic regression analysis. We concluded that RDW was an independent correlate of HCV-related cirrhosis (Table 3).

## 4. Discussion

During the first decade of infection, hepatitis C is characterized by slow progress and no obvious symptoms. By the time the apparent liver-related symptoms appear, the infection would have already reached the advanced stage [14]. HCV infection is more likely to develop into cirrhosis and HCC than hepatitis B virus (HBV) infection. Liver biopsy is a gold standard method for assessing hepatic fibrosis severity. However, liver biopsy is an invasive examination that requires the consideration of contraindications, such as the presence of ascites and severe bleeding tendency in patients, and it may not facilitate dynamic analysis.

RDW is one of the parameters that are used to carry out the morphological classification of anemia. Recently, markers obtained from or calculated using CBC indices, including RDW and/or RPR, were also considered as independent risk factors for studying liver diseases such as HBV-related diseases [8, 10, 15–17], PBC [11], and NAFLD [18]. However, only a few studies in the literature have investigated the relationship between RDW, RPR, and PLR and hepatitis C.

From their study, Lou et al. suggested that the RDW values in acute hepatitis B, chronic hepatitis B, and chronic severe hepatitis B patients were significantly higher than in healthy controls. In addition, they indicated that RDW was associated with the severity of hepatitis B in patients and could independently predict a 3-month mortality rate [8]. Huang et al. found that the RDW values in patients with HBV-related liver disease were significantly higher than in healthy individuals and considered that RDW could be an independent risk factor for estimating the disease severity [17]. The RPR could also be used as an indicator predicting the presence of significant fibrosis and cirrhosis in chronic HBV patients [15].

In their study, Taefi et al. found the prevalence of hepatitis C to be high in patients with various etiologies of chronic hepatitis, including chronic HBV, chronic HCV, alcoholic hepatitis, and PBC [19]. They suggested that RPR was related to the severity of fibrosis and cirrhosis in chronic hepatitis patients. Another study showed that the RDW values in patients with HCV-related cirrhosis were significantly higher than in chronic HCV patients and healthy controls, respectively [20]. They found that the RDW values were positively correlated with Child-Pugh and Model for End-Stage Liver Disease scores. In our study, similar to the study by Jin et al., RDW values in HCV-related cirrhosis patients were found to be significantly higher than those in chronic HCV patients. APRI and FIB-4 are markers used for assessing fibrosis in chronic HCV patients [7, 21, 22], and we found that the RDW and RPR were positively correlated with these two parameters in this study. PLR was negatively correlated with FIB-4. Furthermore, RPR and 1/PLR values were found to be significantly high in HCV-related cirrhosis. The ROC curves of RDW, RPR, and 1/PLR showed excellent AUCs.

The RDW has been considered to be independently associated with inflammatory markers in a large cohort of unselected outpatients [23]. Similar to other chronic diseases cases, patients with chronic HCV infection have the nutritional deficiencies [24]. Increased RDW may be associated with chronic inflammation and nutritional deficiency [25]. In addition, elevated RDW is closely associated with hemolytic anemia in HCV-related liver disease [24]. Furthermore, HCV-infected patients can potentially have thrombocytopenia, and its degree may be associated with the disease severity [26, 27]. Platelet count was also considered to be negatively correlated with hepatic fibrosis [15]. For these reasons, a relative increase in RPR and decrease in PLR may occur in patients with HCV infection.

## 5. Conclusion

In conclusion, RDW, RPR, and PLR can be considered along with other biomarkers that indicate the severity of HCV infection in patients. Certainly, RDW, RPR, and PLR can be used as noninvasive, fast, convenient, and real-time monitoring markers in HCV-infected patients.

## Figures and Tables

**Table 1:** Comparison of demographic and laboratory results between HCV-infected patients and controls.

Parameters	HCV-infected patients (*n* = 94)	Controls (*n* = 84)	*P* value
Gender (male/female)	65/29	57/27	0.853
Age (years)	47.23 ± 12.78	44.98 ± 8.38	0.304
WBC count (×10^9^/L)	5.61 ± 2.22	6.59 ± 1.32	<0.001
Hemoglobin (g/L)	121.02 ± 27.69	135.14 ± 19.06	<0.001
Platelet count (×10^9^/L)	124.84 ± 66.14	257.72 ± 57.64	<0.001
RDW (%)	15.18 ± 2.59	13.39 ± 1.17	<0.001
RPR	0.18 ± 0.14	0.05 ± 0.01	<0.001
1/PLR	0.018 ± 0.010	0.010 ± 0.004	<0.001

Data are expressed as *n* or mean ± SD. HCV: hepatitis C virus; RDW: red cell distribution width; RPR: red cell distribution width to platelet ratio. PLR: platelet-lymphocyte ratio.

**Table 2:** Comparison of demographic, clinical characteristics, and laboratory results between chronic HCV and HCV-related cirrhosis patients.

Characteristics and parameters	Chronic HCV (*n* = 52)	HCV-related cirrhosis (*n* = 42)	*P* value
Gender (male/female)	36/16	29/13	0.985
Age (years)	45.31 ± 14.28	49.51 ± 10.45	0.103
WBC count (×10^9^/L)	6.21 ± 1.84	4.89 ± 2.49	0.004
Hemoglobin (g/L)	134.18 ± 15.77	105.41 ± 30.68	<0.001
Platelet count (×10^9^/L)	167.68 ± 52.51	74.03 ± 39.17	<0.001
Total bilirubin (*μ*mol/L)	13.89 ± 8.72	35.13 ± 44.85	<0.001
Total protein (g/L)	68.14 ± 7.69	66.34 ± 8.94	0.297
Albumin (g/L)	36.19 ± 5.18	29.89 ± 5.39	<0.001
AST (U/L)	57.75 ± 36.56	88.39 ± 101.06	0.112
ALT (U/L)	86.67 ± 75.59	80.67 ± 126.06	0.777
AAR	0.89 ± 0.43	1.33 ± 0.49	<0.001
PT (s)	11.15 ± 1.03	14.36 ± 4.14	<0.001
INR	0.95 ± 0.08	1.31 ± 0.41	<0.001
Creatinine (*μ*mol/L)	76.89 ± 21.94	88.07 ± 87.38	0.381
RDW (%)	14.12 ± 1.54	16.44 ± 3.01	<0.001
APRI	0.85 ± 0.57	3.08 ± 3.31	<0.001
FIB-4	2.07 ± 1.31	7.94 ± 4.91	<0.001
RPR	0.09 ± 0.04	0.28 ± 0.15	<0.001
1/PLR	0.022 ± 0.011	0.015 ± 0.009	0.002
HCV RNA (IU/mL)	9.95 × 10^6^ ± 2.49 × 10^7^	7.79 × 10^6^ ± 1.48 × 10^7^	0.605

Data are expressed as *n* or mean ± SD. HCV: hepatitis C virus; AST: aspartate aminotransferase; ALT: alanine aminotransferase; AAR: aspartate aminotransferase to alanine aminotransferase ratio; PT: prothrombin time; INR: international normalized ratio; RDW: red cell distribution width; APRI: aspartate aminotransferase-to-platelet ratio index; FIB-4: fibrosis index based on the 4 factors; RPR: red cell distribution width to platelet ratio; PLR: platelet-lymphocyte ratio.

**Table 3:** Risk factors associated with the presence of cirrhosis in patients with chronic HCV infection.

	*β*	SE	Wald	*P* value	OR	95% CI
Age (years)	−0.072	0.044	2.673	0.102	0.931	0.854–1.014
AAR	0.085	1.130	0.006	0.940	1.089	0.119–9.981
APRI	−0.510	0.372	1.880	0.170	0.600	0.289–1.245
FIB-4	1.522	0.497	9.363	0.002	4.579	1.728–12.135
RDW (%)	0.402	1.187	4.618	0.032	1.494	1.036–2.155

OR: odds ratio; CI: confidence interval. AAR: aspartate aminotransferase to alanine aminotransferase ratio; APRI: aspartate aminotransferase-to-platelet ratio index; FIB-4: fibrosis index based on the 4 factors; RDW: red cell distribution width.

## Abbreviations

HCV: Hepatitis C virus
RDW: Red cell distribution width
RPR: Red cell distribution width to platelet ratio
PLR: Platelet-lymphocyte ratio
AST: Aspartate aminotransferase
ALT: Alanine aminotransferase
AAR: Aspartate aminotransferase to alanine aminotransferase ratio
PT: Prothrombin time
INR: International normalized ratio
APRI: Aspartate aminotransferase-to-platelet ratio index
FIB-4: Fibrosis index based on the 4 factors
ROC: Receiver operator curves.
